# Microalgae Biomass as a Potential Feedstock for the Carboxylate Platform

**DOI:** 10.3390/molecules24234404

**Published:** 2019-12-02

**Authors:** Jose Antonio Magdalena, Cristina González-Fernández

**Affiliations:** Biotechnological Processes Unit, IMDEA Energy, 28040 Madrid, Spain; joseantonio.magdalena@imdea.org

**Keywords:** anaerobic fermentation, carboxylate platform, microalgae, microbial communities, operational conditions, volatile fatty acids

## Abstract

Volatile fatty acids (VFAs) are chemical building blocks for industries, and are mainly produced via the petrochemical pathway. However, the anaerobic fermentation (AF) process gives a potential alternative to produce these organic acids using renewable resources. For this purpose, waste streams, such as microalgae biomass, might constitute a cost-effective feedstock to obtain VFAs. The present review is intended to summarize the inherent potential of microalgae biomass for VFA production. Different strategies, such as the use of pretreatments to the inoculum and the manipulation of operational conditions (pH, temperature, organic loading rate or hydraulic retention time) to promote VFA production from different microalgae strains, are discussed. Microbial structure analysis using microalgae biomass as a substrate is pointed out in order to further comprehend the roles of bacteria and archaea in the AF process. Finally, VFA applications in different industry fields are reviewed.

## 1. Introduction

Under the Europe 2020 growth strategy, the European Union (EU) is currently updating its legislation to promote a shift to a more sustainable model known as a circular economy [1]. The use of waste streams and renewable resources appear as a core priority to reduce the actual carbon footprint of the state members. These directives prioritize the development of efficient alternatives to the traditional fossil fuels employed for energy and commodity generation. Nowadays, one of the investigation lines gaining importance is the use of microbial consortia to produce high value added products such as carboxylates (volatile fatty acids, VFAs) through anaerobic fermentation (AF), mostly known as the carboxylate platform [2,3]. Traditionally, anaerobic digestion (AD) converts complex substrates into biogas, containing methane (bioenergy) and a digestate. However, this new approach involves the conversion of biomass to bulk chemicals (bioproducts), which is economically more profitable than biogas production [4,5]. Acetic, propionic, (iso)butyric, (iso)valeric and caproic acids are VFAs traditionally obtained through the petrochemical pathway. These compounds can be further used as building blocks in different fields of the industry including food additives, pharmaceuticals, adhesives, solvents or chemical intermediates [6,7].

Among the feedstocks that can be subjected to AF, microalgae biomass arises as a potential alternative for VFA production. It should be highlighted that this biomass can be valorized for high value bioproducts instead of being produced specifically for VFA generation. More specifically, one of the weaknesses of microalgae-based bioproduct production is the nutrients required. However, when this technology is combined, for instance, with wastewater bioremediation by photosynthetic means, the overall balance becomes positive [8]. When cultivated in this manner, biomass cannot be used for nutraceutical, feed or food purposes. Moreover, the harvested biomass is normally poor in fermentable sugar or transesterificable lipids, and thus, the most straightforward use of algal biomass is AD.

VFA production requires a revisit of the AD process for better comprehension of the overall process as this technology has been traditionally used for biogas production. In this sense, there are different variables that deserve further study, such as (i) the substrate employed, (ii) the operational conditions imposed, and (iii) the developed microbiome within the bioreactor. The use of waste streams is cost-effective, and also helps decrease the overall process costs as it contributes to residues management. However, microorganisms in AD are often not able to directly utilize complex organic matter. Hence, a pretreatment step is needed prior to digestion by using physical, chemical, or enzymatic methods to increase the soluble organic matter availability [9,10]. Additionally, there is a need to consider factors related to the operational conditions in the system. Temperature, inoculum used, retention times (solid and hydraulic), organic loading rate (OLR) and pH directly affect VFA production and profiles [11,12]. Finally, the existing bacterial populations must assure a good conversion of organic matter into VFAs. For such a goal, it is considered crucial to inhibit the methanogenic population to accumulate VFAs. As a matter of fact, archaea activity is linked to secondary syntrophic carboxylate-oxidation reactions of propionic and butyric acids to acetate and hydrogen, reducing the amount of VFAs in the digestate [3].

The aim of this work is to review the potential advantages of waste streams as feedstock for VFA production, paying specific attention to microalgae biomass. In addition, the operational conditions and the microbiome related to the acidogenic stage of the AD will be reviewed.

## 2. Volatile Fatty Acid Production by Means of Anaerobic Fermentation 

**Figure 1:**
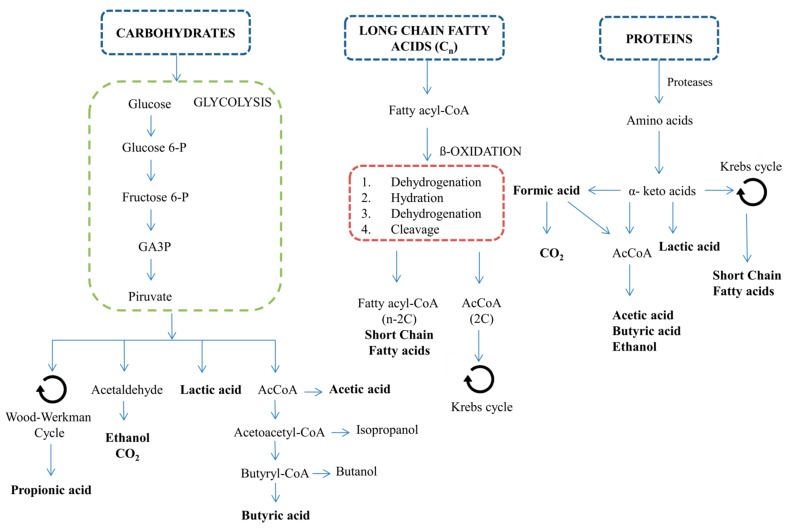
A simplified overview of metabolic pathways involved in VFA synthesis from the main macromolecules of microalgae biomass. GA3P, glyceraldehyde-3-phosphate; AcCoA, Acetyl-CoA.

Volatile fatty acids (VFAs) are formed during the fermentative stages (acidogenesis and acetogenesis) of the AD process (Figure 1). These chemicals are very versatile as building blocks and are commonly used in different industrial processes. The range of applications is quite broad [7,13]. For instance, acetic acid has an important role in the food industry [14], propionic acid is mainly used as an acidifier for animal feed and grain [15], and butyric acid can be utilized as a precursor for biofuel production [16]. Therefore, VFA production through biological catalysis in the AF process is a very promising technology due to its wide range of applications. VFA production through AF occurs at milder temperature and pressure conditions than petrochemical pathways, which is beneficial in terms of energy consumption [17].

**Figure 2:**
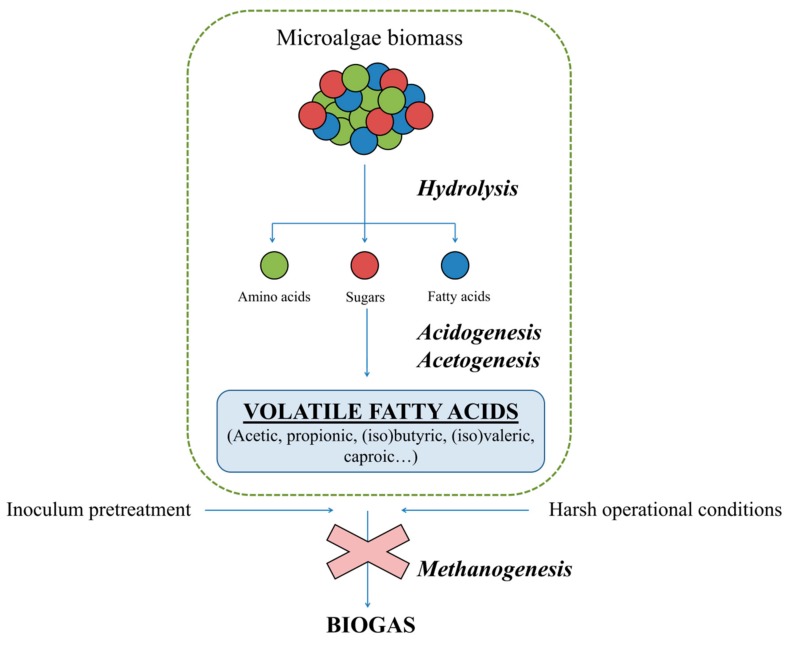
The use of microalgae biomass as feedstock for the carboxylate platform.

AD is a robust and well-known process, and a wide variety of substrates can be subjected to this technology regardless of their macromolecular composition. The substrate chosen undergoes four different steps; hydrolysis, acidogenesis, acetogenesis and methanogenesis. Firstly, exo-enzymes belonging to hydrolytic bacteria degrade the complex organic matter composed of carbohydrates, proteins and lipids into their respective monomers namely, sugars, amino acids and long chain fatty acids. The efficiency of this stage often conditions the overall process yields, as it determines the total soluble organic matter availability. Secondly, the acidogenic bacteria anaerobically oxidize the soluble monomers originating from the VFAs, CO_2_, alcohols, H_2_, and lactic acid. Afterwards, acetic acid, CO_2_ and H_2_ are produced by acetogenic bacteria. These products are the main substrates for the methanogenic archaea, which are in charge of the methanogenic stage. These microorganisms can be classified into two different groups depending on the substrate metabolized for biogas production. Acetoclastic archaea use acetic acid to produce methane, whereas hydrogenotrophic microorganisms use H_2_ and CO_2_ as the main substrates to obtain methane. The inhibition of this latter step of the AD is considered crucial as, otherwise, VFAs would be degraded and finally transformed into biogas (Figure 2).

### 2.1. Microalgae Biomass as a Substrate for VFA Production

The selection of a cost-effective substrate for VFA production is of paramount importance to decrease the overall production cost. Up to date, different sugar-based carbon sources have been tested for VFA production [18,19]. However, cheaper substrates such as waste streams could be ideally used to reduce the costs of the process. Microalgae, for instance, can be considered a residual stream when grown in wastewater. Microalgae-based systems for wastewater treatment have been shown to be a promising technology [8,20]; however, the biomass generated can be further processed into something more valuable than biogas.

The physical state and composition of a substrate directly affect the hydrolysis efficiency and hence, the VFA conversion yields. In this sense, microalgae biomass is considered a complex substrate and that is why recent research efforts have been conducted to improve the hydrolysis step [9]. Different pretreatment methods (e.g., thermal, mechanical, chemical or biological) have been proven to increase the solubilization of the organic matter by means of cell wall disruption/hydrolysis. These pretreatment techniques have been widely studied for biogas production using microalgae biomass [17,21]. These methods could be an interesting option to improve VFA production from microalgae biomass.

In addition to the cell wall protecting microalgae cells, another important aspect is the macromolecular distribution of the substrate that can be classified regarding its content in carbohydrates, proteins and lipids. With regard to microalgae biomass, the amount of each macromolecule appears to be very variable depending on the growth conditions and the strain assessed [22]. However, in general terms, proteins are the most abundant macromolecule of green microalgae, accounting from 30 to 60% of their dry weight [23]. The AD of a protein-rich substrate might constitute a drawback for biogas production. Indeed, this macromolecule has been shown to hamper biogas production when using protein-rich microalgae biomass [9,24]. Nevertheless, it may turn out as an attractive feedstock characteristic when AF is directed towards VFA production. The first stage of the AD entails the hydrolysis of the complex macromolecules composing the microalgae biomass. During this phase, the protein fraction is cleaved into simple amino acids and releases the nitrogen contained in the form of ammonium (NH_4_^+^), and free ammonia (NH_3_). The amount of each species relies on pH and temperature conditions. High amounts of these compounds [25] are often associated with digestion failure when targeting biogas production due to the inhibition of the methanogenic step [26,27]. Therefore, this methanogenic weakness towards NH_4_^+^ and NH_3_ seen in biogas production might in fact represent an advantage for VFA generation, as the inhibition of this microbial community would contribute to VFA accumulation.

Proteins, carbohydrates and lipids present different hydrolysis rates [28]. As a result, different VFA conversion yields and profiles can be obtained depending on the substrate composition (Table 1). Results collected in Table 1 suggest that besides the macromolecular composition of the microalgae biomass, there are other factors affecting VFA production and profiles, such as the operational conditions established and the microorganisms carrying out the biological process. 

### 2.2. Operational Conditions for VFA Production

The manipulation of process variables such as inoculum, pH, temperature, HRT and OLR have a great influence not only on VFA accumulation, but also on the obtained VFA profiles [11,12]. This is because these operational conditions ultimately affect the delicate relations among microbial populations. Methanogenic archaea are more sensitive to operational changes than organic acid producers [39]. As it was aforementioned, archaea are the main organisms responsible for VFA consumption, and thus, their inhibition is considered of paramount importance for attaining competitive VFA yields.

#### 2.2.1. Inoculum

Microorganisms present in the anaerobic sludge are very diverse as many species are involved in the AD process. When selecting microalgae biomass for AD, it is important to take into account the interactions with the anaerobic microbiome. For instance, marine microalgae species, such as *Isochrysis galbana*, *Dunaliella salina* or *Nannochloropsis salina,* have been proposed for AD for biogas production [40,41,42]. These strains hinder the AD process due to high salinity causing plasmolysis in the anaerobic populations (VFA producers and archaea) due to high external osmotic pressure. A long acclimation period for the inoculum, the use of compatible solutes and the employment of halophilic populations are regarded as strategies to be applied to the inoculum to overcome these issues in order to be able to use these species as substrates.

Each stage of the AD process is characterized by different groups of microorganisms. Among others, organic acid-producing bacteria are distinguished during the fermentative stages (hydrolysis and acidogenesis), and methanogenic archaea during methanogenesis. The species involved use different molecules as substrates, and release different products according to their metabolism, resulting in a complex scheme of reactions and products. Therefore, when VFA production is desired, reduction of methanogenic archaea in the inoculum is appropriate to avoid VFA consumption. Strategies applied to the inoculum, such as thermal pretreatments and the addition of chemicals, have been tested. Thermal pretreatment implies subjecting the inoculum to high temperatures during determinate periods of time with the aim of eliminating non spore forming microorganisms. This type of pretreatment has been applied in the literature to substrates other than microalgae [43,44]. A mixture of *Desmodesmus* sp., *Scenedesmus* sp., and *Chlamydomonas* sp. was digested with an anaerobic inoculum subjected to a thermal pretreatment (100 °C for 2 h) to inactivate methanogens and the results showed organic matter conversion into VFAs up to 50% VFAs-COD/CODin at 55 °C [35]. Additionally, pretreatment of inoculum at 120 °C for 10 and 30 min using *C. vulgaris* as the substrate rendered organic matter conversion into VFAs up to 71% [45]. On the contrary, low temperature pretreatments in this study promoted biogas production. However, thermal pre-treatments should be conducted in such a way that only methanogens are affected, as conditions that are too harsh can not only eliminate methanogens but also organic acid producers [46].

The addition of chemicals is used to block methanogen enzymes. Different compounds have been used for this goal, such as 2-bromoethanesulfonate (BES), iodoform or chloroform. In this context, BES (50 µmol/mL) prevented methanogenesis when microalgae biomass composed of *S. quadricadua* and *C. vulgaris* was used for VFA production [33]. This trend was maintained when treating an inoculum with BES (10 and 30 mM) [45]. No methane was detected and VFAs accumulated up to 50% VFAs-COD/CODin. In addition, iodoform (30, 50 and 70 ppm *v*/*v*) inhibited methanogens, causing VFA accumulation, when *Laminaria japonica* was employed as a substrate in an AD process (35 °C and pH 6.5–7) [47]. VFA concentration (8 g/L VFAs) was maximized when using 50 ppm of iodoform, whereas further increases negatively affected VFA productions (70 ppm, reported values similar to those found in the control, 6 g/L), suggesting the negative effect of iodoform on the rest of the microbiome.

In general, the use of chemicals and thermal pretreatments applied to the inoculum are able to inactivate methanogens. Nevertheless, the high prices, the environmental concerns, and the high energy input requirements are the main drawbacks. In addition, these strategies often show short-term effects on the continuous operation towards methanogens and thus, other methods (manipulation of operational conditions) are recommended for VFA accumulation.

#### 2.2.2. pH

pH influences the growth rate of the fermentative microorganisms in charge of VFA production and the optimum enzymatic activities during the hydrolytic step. Moreover, each group of microorganisms has an optimum pH working value. Whereas methanogenic archaea grow better at a pH close to neutrality, acidogenic and hydrolytic bacteria have a wider pH growth range. Previous studies have estimated the optimum range for the acidogenic bacteria at around 5 to 7 [19,48]. Investigations regarding the effect of pH on VFA productions from microalgae biomass showed different results, most likely due to the wide range of microalgae strains and operational conditions employed. For instance, digestion of *Chlorella* sp. at acidic pH values (5.5) and 25 °C showed up to 47% VFAs-COD/CODin, similar to what was attained in the same experiment at neutral pH values and the same temperature (7.5, 45.1% VFAs-COD/CODin) [30]. However, the use of pH values in the basic range has also resulted in good organic matter conversions into VFAs. The digestion of *Microcystis* at pH 10 and 25 °C retrieved an organic matter conversion into VFAs of 31.5% VFAs-COD/CODin. In this sense, some authors pointed to the higher hydrolysis rates achieved at basic pH values as the reason for the conversion yield attained [49]. This feature was also observed when using other protein-rich substrates [50]. Both studies gave acetic and propionic acids as the main fermentation products. Other investigations using microalgae biomass as a substrate for VFA production followed this trend (Table 1).

#### 2.2.3. Temperature

Similar to pH, temperature affects not only the metabolism of the microorganisms and their enzymatic activities, but also the physical state of the organic matter. In this manner, temperature is positively correlated with organic matter solubilization and determines the development of certain microbial communities impacting VFA production and profiles. High fermentation temperatures (50 °C) resulted in high conversion yields (40% VFAs-COD/CODin) when non-pretreated *Chlorella* sp. was digested at pH 6.4, while the use of 25 °C and 35 °C mediated lower conversions (17 and 38% VFAs-COD, respectively) [29]. On the contrary, other investigations obtained similar conversion yields (45–48% VFAs-COD/CODin) at temperatures of 25 °C and 35 °C when compared to 50 °C (37% VFAs-COD/CODin) when using protease pretreated *Chlorella* sp. as a substrate [30]. These differences might rely on the state of the biomass (raw or pretreated). Whereas Magdalena et al. (2018) used a proteolytic pretreatment, Kim et al. (2019) did not hydrolyze the biomass prior to AD. Hence, the high temperatures at which this latter investigation was conducted most likely increased biomass solubilization, and thus, VFA yields, at the highest temperature. With regard to VFA profiles, acetic and propionic acids were the most abundant products regardless of the temperature employed and the butyric acid fraction gained importance at higher temperatures in these experiments [29,30].

#### 2.2.4. Organic Loading Rate (OLR)

The organic loading rate (OLR) is the amount of organic matter present in the substrate applied to a certain volume of media per unit of time. OLR selection is process specific and has been studied previously for biogas production [51]. The general trend observed with other substrates is an increasing VFA production at stepwise OLR increases [52]. VFA accumulation leads to a drop in pH and a final decay of methanogens. Nevertheless, it is also true that there is a maximum OLR threshold where no further improvements are obtained. This fact might be explained by taking into account the hydrolytic stage of the AD process. When reactors are fed at high OLR (values), the hydrolytic capacity of the system is exceeded, and thus, the process becomes unstable and no further improvement is noticed. A recent study analyzing the effect of stepwise OLR increases (3, 6, 9, 12, 15 g COD/Ld) for VFA production using *C. vulgaris* as a substrate revealed an optimum VFA production at 12 g COD/Ld (0.37 ± 0.02 COD-VFAs/CODin) with respect to the highest OLR (0.29 ± 0.01 COD-VFAs/CODin) [53]. Authors used a proteolytic pretreatment to overcome hydrolytic deficiencies and highlighted that the bottleneck of the process was found to be the acidogenic stage, most probably due to the combined effect of high ammonium, VFAs and Na^+^ concentrations.

#### 2.2.5. Hydraulic Retention Time (HRT)

The hydraulic retention time (HRT) is a design parameter that establishes the time that the organic matter remains in the reactor. It is closely related to the OLR selected for the process. At low HRT values, microorganisms exhibiting low growth rates are possibly washed out from the reactor as they do not have enough time to grow and adapt to the harsh environmental conditions. These conditions may provoke a drop in the species diversity present in the system as certain species are not able to grow [38]. On the contrary, when HRT values are high, more populations are likely to grow and take part in the AD process. In this context, methanogenic archaea have been reported to exhibit lower growth rates than acidogenic bacteria [54]. Therefore, this parameter could be used as a tool to select the most suitable populations in charge of organic acid accumulation, as the use of low HTR could favor the wash out of methanogens. However, values for HRT must be high enough to allow the anaerobic microorganisms to carry out the hydrolysis and acidogenesis of the substrate. For instance, the use of low HRT favored VFA production in a semi-continuous bioreactor fed with *C. vulgaris* in which the use of HRT for 8 days showed higher productivities than 10 and 12 days, most probably because of a better activity of methanogens at higher HRTs [38]. This fact means that maximum conversion yield can be achieved in a shorter period of time than that needed for biogas production with a direct impact on the reduction of the total economic process costs. For instance, HRT of 15 and 20 days has been used for the AD of *C. vulgaris* biomass for biogas production [27], while the HRT can be reduced to 8 days for VFA accumulation purposes.

Therefore, considering the high heterogeneity of microalgae strains and macromolecular composition, even among the same species, as well as the different operational conditions imposed on the system, it is only possible to set approximate guidelines for maximum conversion of organic matter into VFAs.

## 3. Microbial Populations Involved in VFAs Productions 

Microbial populations present in an anaerobic inoculum have a determining influence on the AD performance. Each AD stage presents different microorganisms (hydrolytic and fermentative bacteria) during hydrolysis, acidogenesis, acetogenesis and (methanogenic archaea) during methanogenesis. The relative abundance of species during AD might affect the fate of the organic matter. In this sense, manipulation of operational conditions could promote organic acid producers and inhibit those microorganisms in charge of methanogenesis to enhance VFA accumulation. In addition, the study of microbial populations can shed light on AD assigning roles to microbial populations.

Microbial structure is widely dependent on the AD final goal, either biogas or VFA production. This difference is caused by the operational conditions imposed on the system (Section 2.2) resulting in a sludge specialization. In this sense, a recent study regarding the metagenome for biogas generation highlighted the high flexibility, diversity and adaptability to operational conditions and substrates of the anaerobic community [55]. Opposite to that, reactors involved in VFA production are often less diverse and exhibit different species than those devoted to biogas production.

With respect to the bacterial community, Firmicutes, Proteobacteria and Bacteroidetes have been identified as the major contributing phyla in anaerobic fermenters devoted to VFA productions. These phyla have been claimed to produce VFAs as well as actively degrade proteins and polysaccharides, which in fact represent a high percentage of the macromolecular distribution of microalgae biomass (Table 1) [56]. The PCR-DGGE analysis carried out at different temperatures (35, 45 and 55 °C) when microalgae biomass was digested for VFA production displayed a clear dominance of microbial species belonging to Firmicutes, Proteobacteria and Bacteroidetes [35]. Furthermore, this investigation also concluded that diversity decreased at the highest temperature, in which VFA production achieved the highest conversion (COD-VFAs/CODin). Following this trend, Proteobacteria (65.7%) and Firmicutes (29.0%) were dominant when activated carbon was used to enhance VFA production from *Microcystis* [31]. Likewise, species belonging to Firmicutes were the most abundant (45–70% in terms of relative abundance) followed by Bacteroidetes (10–35%) when cyanobacterial biomass was digested for VFA production [57]. A similar investigation also remarked on the presence of the Firmicutes phylum with species such as *Sporanaerobacter acetigenes* (13%) or *Soehngenia* (12%), identified as the major VFA producers when the microalgae strain *Ettlia* sp. was subjected to AD [58]. All of these investigations were carried out at batch scale; however, when operating semi-continuous fermenters fed with *C. vulgaris*, Fimicutes dominated in the bacterial community [38].

Concerning the Euryarchaeota phylum, these species are detrimental for VFA accumulation [3]. Hence, their inactivation is of high importance in achieving competitive VFA production. According to their metabolism, archaea species can be divided into acetoclastic or hydrogenotrophic. The latter ones are often more robust than the acetoclastic ones [59]. Thus, it is expected that in the case that biogas is produced, reactors devoted to VFA production remove organic matter through the hydrogenotrophic pathway. In fact, hydrogenotropic species such as *Methanobacterium* were reported by Magdalena et al. (2019), while other investigations did not find a significant archaea, acetoclastic or hydrogenotrophic presence [58]. In this particular case, those authors applied daily iodoform (8 mg/L) to suppress any methanogenic activity and this would explain the lack of archaea.

Overall, the reviewed investigations related to the microbial structure of bioprocesses aiming at VFA production are clearly different to the ones using microalgae biomass as a substrate for biogas generation. The relative abundance of each phylum is dependent on the operational conditions established in the reactor. Hence, the use of harsh operational conditions to inactivate methanogens and promote VFA producers results in a sludge specialization, where methanogenic activity is outcompeted by fermentative bacteria. This fact might hamper biogas production and in turn boost VFA accumulation.

## 4. VFAs As Building Blocks for the Industry

VFAs produced from microalgae biomass fermentation might be a product by itself (after separation and purification) or serve as platform molecules for different applications within several fields in the industry. Some of the promising applications that these molecules might encounter include the production of biodegradable plastics such as polyhydroxyalkanoates (PHAs), energy generation from microbial fuel cells (MFCs), VFA elongation into longer fatty acids via reverse ß oxidation and their use as building blocks for oil-based chemistry via oleaginous yeast fermentation.

PHAs are currently produced using microbial isolates and well-defined substrates, which increase overall production costs [60]. However, VFAs produced from waste streams appear as a promising alternative to reduce the price of the process [61]. In this sense, PHAs might be produced from the VFAs present in the digestate obtained after microalgae fermentation. Filtering this broth is advised to remove microorganisms and to control the amount of ammonium and phosphorous to allow PHA production [62]. Results using different fermented wastes as substrates in mixed cultures have resulted in microbial systems exhibiting PHA contents in the range of 40–77% (DW %) [7], whereas other authors have addressed the importance of VFA distribution on final PHA composition [63,64].

Another application might be the electricity generation in MFCs [65]. MFCs are made up of an anode where the biofilm oxidizes the soluble VFAs, producing electrons. This current flows towards the cathode where an electron acceptor is reduced. Recently, this technology has attracted the attention of the scientific community [66,67], but operational conditions still need to be optimized as process yields significantly vary depending on the VFA profiles [68]. As a matter of fact, the investigation conducted by Teng et al. (2010) found a different contribution of acetic, propionic and butyric acids to electricity generation. Those authors attributed electricity generation mainly to the presence of acetic and propionic acids, whereas butyric acid exerted a negative impact [69].

The chain elongation (CE) process transforms short VFAs (C2–C5) into medium carboxylates (C6–C12) [52]. These compounds have more value than biogas or VFAs and can be further used in several fields of the industry (aviation fuels, solvents, lubricants or feed additives) [70]. In addition, C6–C12 organic acids are more hydrophobic than shorter VFAs. This feature makes them more attractive as a product because it facilitates the subsequent recovery step. The CE is catalyzed by an anaerobic microbiome in strict anaerobic conditions in a metabolic pathway called reverse β-oxidation. In this pathway, an acetyl CoA molecule is added to a carboxylate (acetate), finally elongating two carbons at a time. The oxidation of an electron donor such as ethanol, methanol, hydrogen or lactic acid is necessary for this process to take place. The impact of different operational conditions, such as the selected electron donor, methane inhibitor or the substrate used, on medium carboxylate production has been studied [71]. In general, low productivities are attained due to the use of mixed culture fermentations, and thus, the study of the microbiome may serve to enhance process yields.

Finally, VFAs are regarded as low-cost carbon sources for lipid biosynthesis to produce oil-based products [72]. Oleaginous yeasts such as *Yarrowia Lipolytica* or *Cryptococcus Curvatus* can accumulate up to 60% of their dry weight in the form of lipid bodies [73]. The use of VFAs obtained from waste such as microalgae can help decrease the overall process production costs mainly impacted by the high price of current substrates [74]. The similar characteristics of plant and microbial oils (similar fatty acids profile) make microbial oil production a promising biotechnological tool for biofuel and bioproduct generation. 

## 5. Conclusions

Overall, the use of the carboxylate platform from microalgae biomass might be useful for added-value product generation as well as a feasible technology for proper waste management. Microalgae biomass arises as a potential feedstock for bio-based VFA productions. The effect of operational conditions on VFA production was reviewed. There are yet no optimum operational conditions for VFA production considering the amount of microalgae strains and conditions employed and thus, further investigation is still needed. To fully understand how these variables influence VFA production and profiles, a possible approach might be to direct attention towards the microbial communities developed during the reactor operation. As a matter of fact, operational conditions are interconnected with the microbiome and hence, the study of the combined effect might result in valuable information for VFA production from microalgae biomass.

## Figures and Tables

**Table 1:** Overview of microalgae strains employed in batch and semi-continuous mode for VFA productions: VFA profiles and conversions.

**BATCH MODE**														
**Strain**	**Temperature (°C)**	**pH**	**Pretreatment**	**Composition**	**% DW**	**Ac**	**Pr**	**But**	**IBut**	**Val**	**IVal**	**Cap**	**COD-VFAs/CODin (%)**	**Reference**
	15	6.4		Carbohydrates	47.5	70	30	0	-	-	-	-	17.37	[29]
*Chlorella vulgaris*	35		-	Proteins	20.4	70	20	10	-	-	-	-	38.17	
	55			Fatty acids	0.9	70	10	20	-	-	-	-	40.47	
	15	6.4		Carbohydrates	28.6	70	30	0	-	-	-	-	9.44	[29]
*C. vulgaris*	35		-	Proteins	56.8	50	40	10	-	-	-	-	33.40	
	55			Fatty acids	0.004	60	10	30	-	-	-	-	42.03	
	25			Carbohydrates	25	41	28	7	9	7	8	-	47.7	[30]
*Chlorella* sp.	35	5.5	Enzymatic (proteases)	Proteins	64	26	35	9	12	8	9	-	39.1	
	50			Lipids	10	33	11	15	14	-	27	-	34.5	
	25			Carbohydrates	25	54	21	6	6	6	7	-	45.1	[30]
*Chlorella* sp.	35	7.5	Enzymatic (proteases)	Proteins	64	57	21	6	8	1	7	-	48.3	
	50			Lipids	10	46	17	12	15	-	9	-	37.1	
			Control	Carbohydrates	6	38	14	36			12	-	13.09	[31]
*Microcystis*	25	10	0.5 Activated carbon (g/L)	Proteins	63	50	13	21.1			15.9	-	31.50	
				Lipids	4									
				Carbohydrates	34									[32]
*Chlorella Pyrenoidosa*	35	6.5	-	Proteins	48	52	11	35	-	-	-	3	4.28	
			140 °C, 10 min 1% H_2_SO_4_	Lipids	18	41	14	37	-	-	-	8	9.14	
*Scenedesmus quadricadua* and *C.vulgaris*	35	5	Non pretreated	NA		40	20	5	20	15	-	-	0.25	[33]
		7.4				50	20	5	10	10	5	-	5.42	
				Carbohydrates	19									[34]
*Arthrospira Platensis*	37	6	2.5% dilute H_2_SO_4_ at 135 °C for 15 min	Proteins	76	40	5	28	5	5	9	8	NA	
				Lipids	5									
	35	6.9		Carbohydrates	NA	86	2	2	0	0	10	0	20.0	[35]
*Desmodesmus* sp., *Scenedesmus* sp., and *Chlamydomonas* sp	45		-	Proteins	NA	74	8	5	2	0	10	0	33.0	
	55			Lipids	NA	66	15	5	1	2	11	0	50.0	
				Carbohydrates	45.5									[36]
*Ettlia* sp	35	7.2	1% NaOH + ultrasound	Proteins	35	64	25	11	-	-	-	-	25.25	
				Lipids	5.5									
**SEMI-CONTINUOUS MODE**														
**Strain**	**Temperature (°C)**	**Operational conditions**		**Composition (%) DW**		**Ac**	**Pr**	**But**	**Ibut**	**Val**	**Ival**	**Cap**	**COD-VFAs/CODin**	**Reference**
*Scenedesmus* sp. Frozen	35	HRT 15 days OLR 2.5 VS/Ld		Carbohydrates	45	11	3	57	18	3	3	4	0.171 g COD-VFAs/g VSin	[37]
	55			Proteins	44	14	1	48	2	0	0	0	0.088 g COD-VFAs/g VSin	
				Lipids	4									
	35	HRT 10 days OLR 1.5 g COD/Ld				14	36	10	11	11	18	-	25.6	[38]
	35	HRT 10 days OLR 3 g COD/Ld		Carbohydrates	21.6	18	32	12	10	11	17	-	25.8	
*C. vulgaris* Enzymatic pretreatment	25	HRT 10 days OLR 1.5 g COD/Ld		Proteins	57.9	20	17	9	17	15	12	9	35.4	
	25	HRT 12 days OLR 1.5 g COD/Ld		Lipids	13.4	24	16	8	20	14	18	13	38.0	
	25	HRT 8 days OLR 1.5 g COD/Ld				24	15	8	20	14	18	12	39.8	

* All the investigations collected in Table 1 were carried out at the lab scale; COD: Chemical oxygen demand.
